# Study on Muscle Fatigue Classification for Manual Lifting by Fusing sEMG and MMG Signals

**DOI:** 10.3390/s25165023

**Published:** 2025-08-13

**Authors:** Zheng Wang, Xiaorong Guan, Dingzhe Li, Changlong Jiang, Yu Bai, Dongrui Yang, Long He

**Affiliations:** 1 School of Mechanical Engineering, Nanjing University of Science and Technology, Nanjing 210094, China; wangzheng19@njust.edu.cn (Z.W.); lidingzhe@njust.edu.cn (D.L.); jiangchanglong@njust.edu.cn (C.J.); byu101010014@njust.edu.cn (Y.B.); dongrui_yang@njust.edu.cn (D.Y.); 2 Hangzhou Zhiyuan Research Institute Co., Ltd., Hangzhou 310013, China; helong@zy-cs.com.cn

**Keywords:** deep learning, mechanomyography, surface electromyography, back-propagation neural network and BERT, muscle fatigue

## Abstract

The manual lifting of heavy loads by personnel is susceptible to the development of muscle fatigue, which, in severe cases, can result in the irreversible impairment of muscle function. This study proposes a novel method of signal fusion to analyse muscle fatigue during manual lifting. Furthermore, this study represents the inaugural application of the back-propagation neural network and bidirectional encoder representation from the transformer (BP + BERT) algorithm to the fusion of two sensor inputs for the analysis of muscle fatigue. Lifting action fatigue tests were carried out on 16 testers in this study, with both surface electromyography (sEMG) and mechanomyography (MMG) signals collected as part of the process. The mean power frequency (MPF) eigenvalues were extracted separately for the two signals, and the results of muscle fatigue labelling according to the trend of the MPF eigenpeak were merged to produce three datasets. Subsequently, the three datasets were employed to categorise muscle fatigue classes using the support vector machine and radial basis function (SVM + RBF), support vector machine and bidirectional encoder representation from transformer (SVM + BERT), back-propagation neural network (BP), and back-propagation neural network and bidirectional encoder representation from transformer (BP + BERT) algorithms, respectively. The results of the muscle fatigue classification model demonstrated that the sEMG and MMG fused dataset, imported into the BP + BERT algorithm, exhibited the highest average accuracy of 98.10% for the muscle fatigue classification model. This study indicates that the fusion of sEMG and MMG signals is an effective approach, and the performance of the BP + BERT muscle fatigue classification model is also enhanced.

## 1. Introduction

Manual lifting of heavy loads usually involves a series of movements such as squatting, reaching, standing upright, turning, and raising the arms. In the absence of protection, manual lifting has problems such as excessive work intensity or improper operation, including irregular lifting movements, which can easily cause muscle fatigue and, in serious cases, lead to acute musculoskeletal injuries and cause local pathological changes. It is necessary to study the degree of muscle fatigue during manual handling, and build a muscle fatigue classification model for manual handling manoeuvres so that the time at which workers’ muscles reach fatigue can be determined and the duration of continuous work can be adjusted to reduce acute musculoskeletal injuries. It is also possible to provide certain evaluation criteria for manual aids to assess whether they can prolong the duration of muscle fatigue in the user.

Muscle fatigue is a temporary decrease in the maximum force of a muscle during contraction, and fatigue occurs in nerve or myofibroblasts [1]. Nutrient depletion and accumulation of metabolites can lead to muscle fatigue, while prolonged muscle activity can also lead to fatigue. Increased awareness of the state of muscle fatigue can help develop a rational exercise programme and avoid muscle damage caused by overexertion. The detection of muscle fatigue is generally based on the processing and analysis of biomedical signals. Biomedical signal processing and analysis is the use of modern information technology to extract various useful information from physiological signals that are masked by noise.

The sEMG and MMG signals contain a lot of information about muscle fatigue. When muscle fatigue occurs, the activity and biochemical properties of the muscle change, resulting in corresponding alterations in the sEMG and MMG signals. The sEMG signal is an electrical signal generated by the activity of nerves and skeletal muscles and is commonly used as a signal source for studying the human muscular system [2]. sEMG signals have been widely used in the fields of rehabilitation medicine, kinesiology, and biomechanics. At present, the research on sEMG signals in muscle fatigue has been in-depth, and many scholars have conducted research on it. It has been shown that during a state of muscle fatigue, the accumulation of lactic acid near the muscle and the increase in electrolytes near the muscle cells lead to changes such as an increase in the amplitude of the sEMG signals [1,2]. The MMG signal is a vibration signal produced by skeletal muscle during contractions. When a muscle is fatigued, the amplitude of contraction of the skeletal muscle is impeded, resulting in an increase in the amplitude of its vibration, which leads to a change in the MMG signals. Jun proposed combining sEMG and MMG signals to detect muscle spasms in patients with brain injury and proved that MMG signals can reflect the state of the muscles [3].

However, the sEMG signal itself has some limitations; when the human body exercises and sweats, it leads to the acquisition of muscle sEMG signals, and cannot accurately judge the muscle fatigue situation. The MMG signal is not affected by this. It has the advantage of easy signal acquisition and easy wear. When the human body performs high-frequency dynamic exercise, the noise of the MMG signal increases due to the addition of interference from external sources of vibration, and the accuracy of judging human muscle fatigue is greatly affected [4]. However, the sEMG signal is less affected by external vibration and has the advantages of accurate signal acquisition and precise muscle position. Under the combined influence of human sweat and external vibration sources, the MMG and sEMG signals can provide a good complementary result.

SEMG signals and MMG signals may correspond to the state of muscle fatigue. However, the specific correspondence between the two signals and muscle fatigue is still the subject of ongoing research. In 2010, Hong-Bo Xie et al. investigated the MMG signal of the biceps brachii muscle in the fatigue state of isometric contraction at 80% of the maximum voluntary contraction level, and the results of their study showed that the MMG signal is a high-dimensional chaotic signal, which can support the analysis and modelling of the MMG signal in fatigue using the nonlinear kinetic theory [5]. In 2015, Hyonyoung Han et al. proposed the feasibility of estimating the muscle contraction force using stiffness measurements under the condition of muscle fatigue, and showed that the muscle contraction force changed by a small amount in the muscle fatigue and non-fatigue states, while the sEMG signal and the MMG signal had a more significant change [6]. In 2016, Isu Shin et al. used MMG signals and a computational geometry technique known as the convex packet algorithm to assess quantitative muscle fatigue during isometric and isotonic contractions, demonstrating that accelerometer-based MMG signals can be used to analyse the electrical activity and low-frequency transverse oscillations of activated skeletal muscle fibres; this can be used to assess muscle function during fatiguing exercise [7]. In 2019, Jannatul Naeem et al. used a support vector machine classifier to classify muscle fatigue states in the spectral cepstrum coefficient features of MMG signals [8]. In 2019, Ibitoye, M. et al. showed that MMG signals can discriminate between different stimulus frequencies and quantify muscle fatigue characteristics during treatment [9]. In 2021, the study by Na Yu et al. aimed to investigate fatigue changes between upper limb muscles during rotational treatment and showed that muscle fatigue characteristics differ at various rotation angles [10]. In 2022, Juan R. M. et al. showed that muscle fatigue can be effectively detected by extracting spectral features of sEMG signals [11].

Several researchers have worked on using machine learning algorithms to classify biosignals for muscle fatigue and human movement. In 2014, G. Venugopal et al. attempted to differentiate between the fatigued and non-fatigued states of sEMG signals using multiple time window features, and demonstrated that the K-nearest neighbour algorithm performed better in classification, achieving a maximum accuracy of 93% [12]. In 2021, Temel S. et al. detected muscle fatigue states in mandibular electromyography (EMG) signals and explored their correlation with bruxism. They used K-nearest neighbour (KNN), SVM, and artificial neural network (ANN) algorithms for classification, achieving a 98.1% classification accuracy for the KNN and SVM algorithms [13]. Also in 2021, Yongqing Zhang et al. proposed a method for detecting muscle fatigue in sEMG signals using a multi-dimensional feature fusion network algorithm. Experimental results showed that this method performed better at predicting muscle fatigue using fused features of different dimensions, achieving an average classification accuracy of 96.45% with the multi-dimensional feature fusion network (MFFNet) algorithm [14].

In 2024, Valentina M. G. et al. de-noised the sEMG signal, extracted the features, and automatically detected the muscle activity status using a signal classification algorithm [15]. In 2024, Nur Achmad S. P. et al. proposed a new method combining feature extraction and deep learning to reduce the effect of feature extraction on EMG pattern recognition [16]. In 2024, Gang Wang et al. developed a noise suppression nulling neural network controller based on the constructed human–computer-coupled model and a convolutional neural network to detect motion intention, which can reduce the error of sEMG signals in joint angle prediction [17].

Existing studies on sEMG signalling and MMG signalling have both been extensive in relation to muscle fatigue; however, one study investigated sEMG signalling in relation to muscle fatigue more than others. In 2022, Weichao Guo et al. proposed a method to comprehensively assess muscle fatigue by fusing microgrid sEMG maps, MMG signals, and NIR spectra. The root mean square and median frequencies of sEMG and MMG signals, blood volume, and muscle oxygenation extracted from NIR spectra were used as fatigue indicators [18]. The study fused the three signals but only used them to increase the data dimensions, and did not discuss and analyse the relationship, strengths, weaknesses, and primary and secondary relationships between the three signals.

Although both signals have certain limitations, they complement each other well. Fusing the two signals can effectively overcome their limitations and make them more useful for muscle fatigue analysis. However, there are few studies on signal fusion, and it is not a prominent area of research despite an increasing number of algorithms being used for fusion. In addition, current deep learning algorithms are being continuously updated, and in general, the updating of the algorithms improves the accuracy of model classification.

This study addresses the issue of the unclear fusion of sEMG and MMG signals and the poor classification accuracy of muscle fatigue classification models. This study proposes a method for integrating the results of the muscle fatigue classification with the trends of the MPF feature peaks of the sEMG and MMG signals during a squatting exercise. Subsequently, the outcomes of the sEMG signal, the outcomes of the MMG signal, and the outcomes following fusion are classified using the BP + BERT deep learning algorithm. The impact of signal fusion is evaluated, and the classification outcomes are compared with those of three algorithms—BP, SVM, and RBF + SVM—to identify the most effective classification algorithm.

## 2. Materials and Methods

### 2.1. Muscle Selection

To study muscle fatigue in manual handling, it is necessary to select the muscles with higher muscle activation. Muscles with higher muscle activation are those that generate more force during movement and tend to show better changes in fatigue state. In a previous study [19], it was found that the following six muscles should be selected in the handling manoeuvre in combination with the muscles that can be supported by the equipment and the muscles with higher muscle activation: the deltoid, obliques, biceps brachii, latissimus dorsi, rectus femoris, and medial femoris muscles.

### 2.2. The Experiment of Muscle Fatigue

The experimenter first squats down on the floor to pick up 10 kg of weight, before resuming an upright position; they then stand up, and during this process cannot bend down in order to prevent a weight that is too heavy for the experimenter’s lumbar injuries, and this continues until the experimenter’s subjective feeling of fatigue stops the experiment.

The subjects were 16 healthy young men, aged 22–26 years, 165–185 cm tall, and 60–80 kg, who had not performed any strenuous exercise for 72 h prior to the test. Training of the test movements was performed prior to the muscle fatigue test collection to ensure the proficiency of the test movements. The ambient temperature of the test environment was set at 24 °C, as human sweat has an effect on the generation of EMG signals. Subjects were warmed up for 5 min to prevent muscle cramps during the experiment.

**Figure 1:**
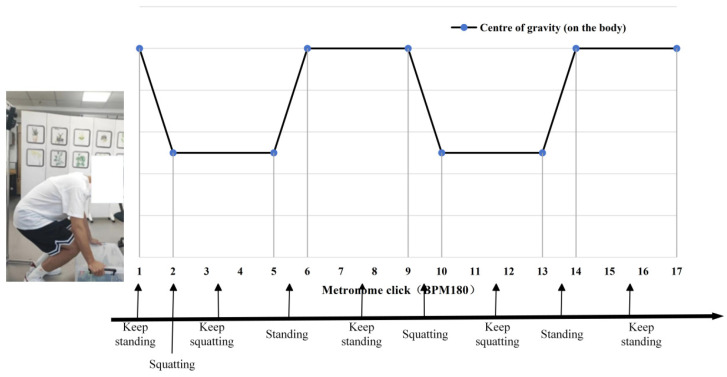
Schematic diagram of the position of the centre of the human body for the experimental movement.

The experimenter performed the handling test according to the metronome rhythm of 180 BPM per minute; the metronome sounded every 4 times for the experimenter to complete a movement, and the movement scheme is shown in Figure 1. A total of 16 datasets were measured in this experiment.

In this experiment, two biosignalling devices were used for simultaneous measurements: the sEMG signal recorded from six muscles on the left side of the body and the MMG signal recorded from six muscles on the right side of the body, allowing both signals to be run simultaneously to measure the experimenter’s movement. The information from the signals collected to select the muscles is shown in Table 1.

**Figure 2:**
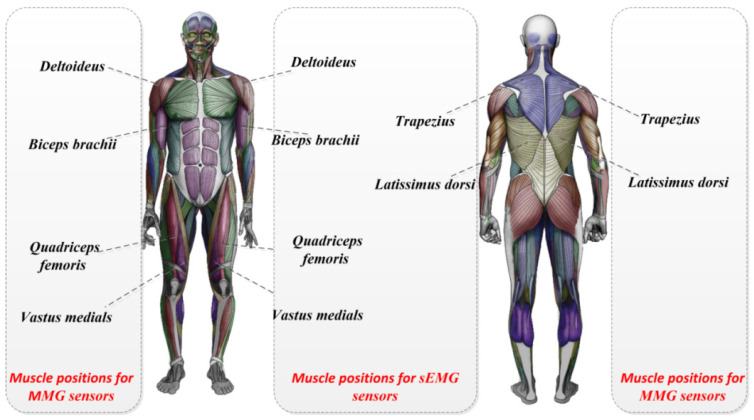
The location of the surface EMG signals or MMG sensors on the muscles.

**Figure 3:**
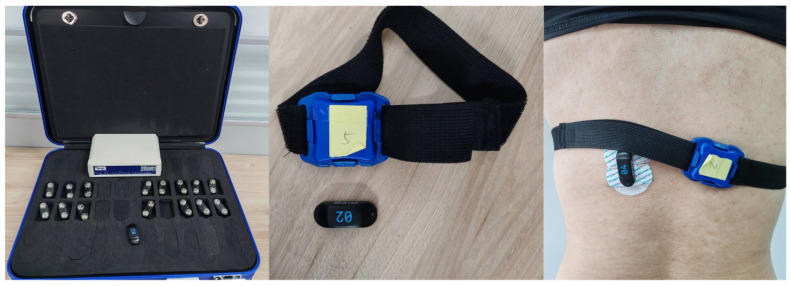
The mounting of physical sensors and human back sensors.

The patch electrodes and the sEMG signal sensors were then attached to the highest part of the muscle belly of the target muscle. The location of the surface EMG signals or MMG sensors on the muscles is shown in Figure 2. The two sensor-mounting locations on the back of the human body are shown in Figure 3.

The experiment followed the ethical guidelines of the Declaration of Helsinki adopted in June 1964 (Helsinki, Finland) and revised in October 2000 (Edinburgh, Scotland). The Ethics Committee of Nanjing Medical University approved the experimental procedures, and the experimental ethical review number was 2021-SR109. All subjects were healthy before participation in the experiment and had no damage to or disease in the muscles tested.

**Figure 4:**
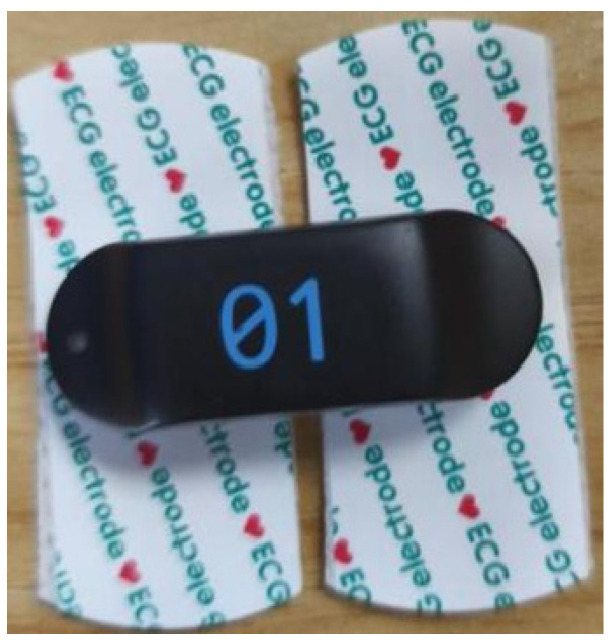
PicoEMG sensor.

In this experiment, sEMG signals were acquired using a Cometa wireless EMG device (a wireless sEMG acquisition device manufactured by Comete, Milan, Italy). The device has a sampling delay of less than 500 us, is equipped with 16 sensors, and has a maximum sampling rate of 4000 Hz. To avoid signal loss, a sampling rate of 2000 Hz was used in this experiment. The experimental sensors are shown in Figure 4.

**Figure 5:**
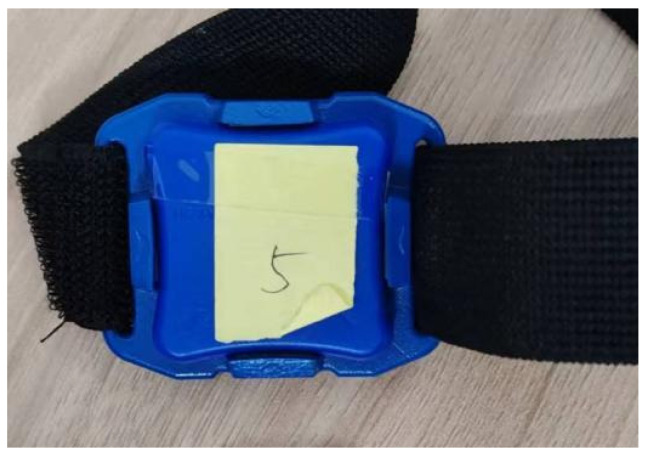
Axis IMU sensors.

The devices used to acquire the MMG signals were 9-axis IMU sensors (LPMS-B2, ALUBI, Guangzhou, China). The sensors are wireless posture sensors that can be used individually or in sets, and in this experiment, six sensors were used with a maximum sampling rate of 400 Hz. The experimental equipment is shown in Figure 5. The MMG signals detect the total muscle changes caused by the activity of the active or stimulated motor unit, which occurs during the transient phases of dynamic contraction or constant-length contraction of the muscle–tendon unit. Thus, when using a posture sensor that detects vibrations on the muscle surface, the vibration detected is the MMG signal [20,21].

**Figure 6:**
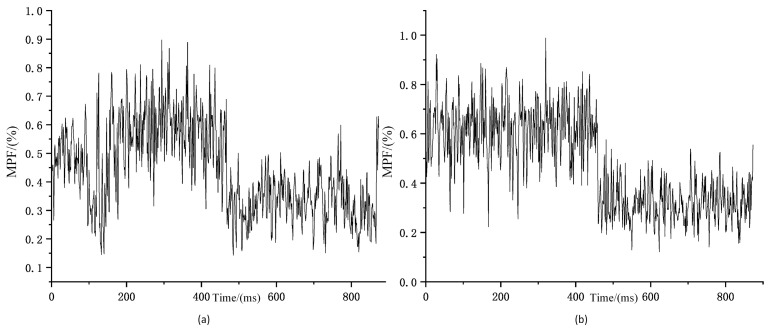
Comparison of trends in MPF changes in bilateral muscles (**a**) Trends in MPF changes of left rectus femoris sEMG signal during squatting exercise; (**b**) Trends in MPF changes of right rectus femoris sEMG signal during squatting exercise.

Since two signals need to be measured simultaneously, the sensors are attached to both sides of the human body for experimental convenience. In related research, it has been proven that the muscles on both sides of the human body have the same trend of change, and after subsequent signal processing normalisation, the signals on both sides can be approximated as the same side of the signal for subsequent analysis. The comparison of SEMG signals from the same muscle on both sides of the human body is shown in Figure 6.

## 3. Theory and Results

### 3.1. Data Pre-Processing and Feature Extraction

**Figure 7:**
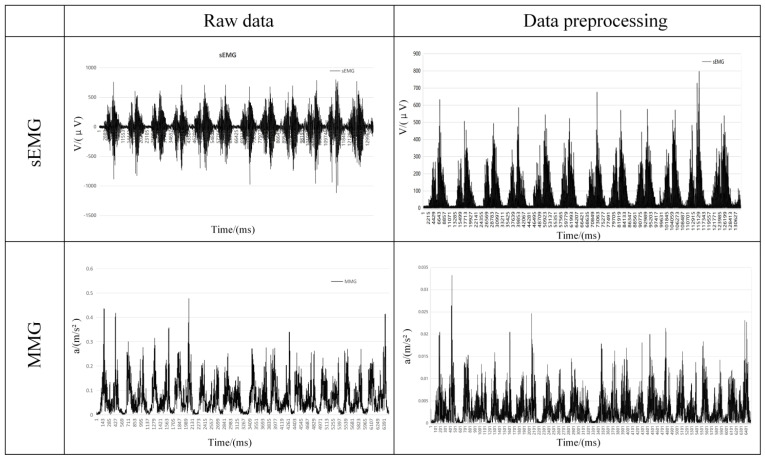
Comparison of two signals before and after pre−processing.

In this study, the sEMG signal was processed for noise reduction. A fourth-order Butterworth filter was used for filtering with a bandpass frequency of 20–500 Hz, and then the signal was corrected after filtering. Since the MMG signal is slightly different in the acquisition frequency, it is a low-frequency signal, and the effective frequency is usually 10–50 Hz, so there is a difference in the bandpass frequency between the two frequencies in pre-processing. In this study, the MMG signal was processed for noise reduction. A fourth-order Butterworth filter was used for filtering with a bandpass frequency of 10–50 Hz, and the signal was corrected after filtering. The before-and-after comparison of the two signals after pre-processing is shown in Figure 7.

After the two signals are pre-processed, feature extraction from the signals is an important part of building a fatigue classification model. A good fatigue classification model is directly related to feature selection. There are three main aspects of feature research on sEMG, including the time-domain feature parameters of EMG signals, such as the integral EMG value and root mean square value; frequency-domain feature parameters, such as the mean power frequency and median frequency; and time–frequency-domain feature parameters, such as instantaneous mean frequency and instantaneous median frequency [22,23]. The complexity of the feature parameter is a relevant description of the complexity of the sEMG signal sequence, and the commonly used complexity feature parameters are complexity, approximate entropy, sample entropy, etc. [24]. Characterisation studies of MMG signals are similar to those of sEMG signals and are mostly included in the above-mentioned eigenvalues.

There are also many features of both signals that correspond to muscle fatigue-related features, including integrated electromyographic values (IEMG), root mean square (RMS), mean power frequency (MPF), median frequency (MF), and so on. However, for the construction of the fatigue classification model, selecting more feature values will increase the mathematical dimension. In this study, two kinds of biosignals were used, with six muscles measured for each, which is already a good data size. Therefore, a synthesis of the existing literature on the characteristics of the two signals revealed that the mean power frequency appears to decrease with deeper muscle fatigue, which is attributed to the decrease in pH, leading to a decrease in the conduction velocity of the muscle fibres [25,26,27]. In this paper, we extracted the MPF eigenvalues of the two signals to analyse them:

(1) $$MPF=\frac{\int_{0}^{\infty }f*PSD\left(f\right)df}{\int_{0}^{\infty }PSD\left(f\right)df}$$

In the above equation, *f* is the frequency of the signal, and *PSD*(*f*) is the power spectral density function of the signal.

(2) $$PSD(f)=\frac{1}{T}{\left|x(k)\right|}^{2}$$

In the above equation, *x*(*k*) is the raw data of the signal.

Since the eigenvalue extraction of the signals is used to label the fatigue state of the muscle movement, in this study, the signals were divided into 120 equal parts, and the eigenvalue solution was performed individually for each segment of the data to better identify the relationship between the eigenvalues and the fatigue state of the muscle.

Since the sEMG signal and the MMG signal have different sampling frequencies and belong to different dimensional data, we needed to normalise the MPF eigenvalues of both, and the normalisation formula is shown below.

(3) $$y_{i}=\frac{x_{i}-x_{\min}}{x_{\max}-x_{\min}}$$

In the above equation, *y_i_* is the result after normalising the eigenvalues, *x_i_* is the MPF data, *x*_min_ is the minimum value in the MPF data, and *x*_max_ is the maximum value in the MPF data.

**Figure 8:**
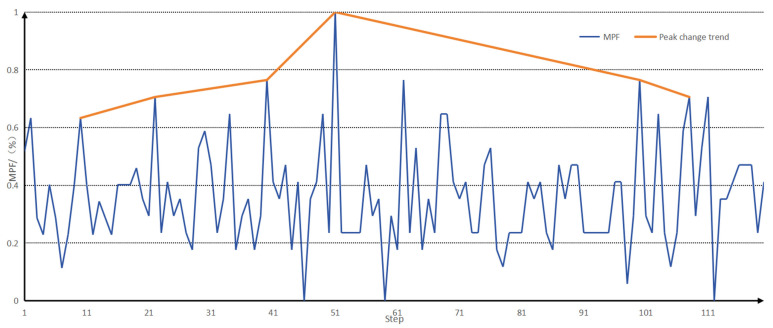
Envelopes of MPF eigenvalues and MPF peaks.

The envelopes of the MPF eigenvalues of the two signals are then drawn for the peaks, and the envelopes of the MPF peaks are shown in Figure 8.

### 3.2. Construction of Datasets

Before importing a muscle fatigue model, it is important to create a muscle fatigue dataset. In this study, feature fusion was performed for MMG and sEMG signals, and the advantages of both signals were combined to improve the accuracy of muscle fatigue detection.

It was found that the peak MPF initially increased with the duration of exercise and then, after reaching the maximum value, the peak MPF gradually decreased. Based on the existing literature [8,9,10], we interpreted the peak MPF after reaching its maximum value as indicating the beginning of the fatigue state. In this study, the change in the MPF peak was divided into the following stages, as shown in Table 2.

Based on the classification in the table above, we labelled the sEMG signal and the MMG signal that was recorded as a change in muscle fatigue state for that muscle. We labelled each muscle separately with the sEMG signal result and the MMG signal result.

**Figure 9:**
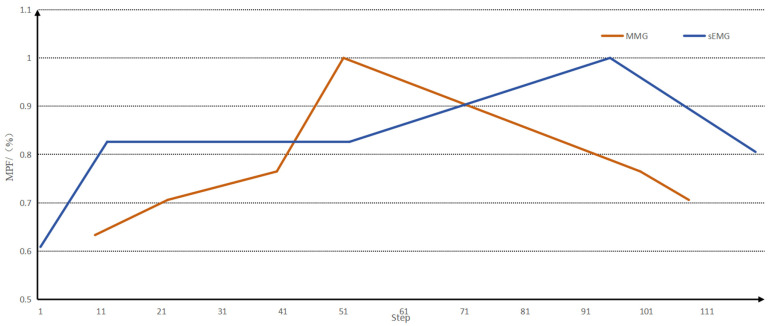
A comparison of the peak envelopes of the MPF eigenvalues of the two signals.

When analysing the fatigue state, the fatigue node of the muscle generally appeared earlier than that of the sEMG signal, but the downward trend of the MMG signal after entering fatigue had a smaller range of variation than that of the sEMG signal, and it was not easy to differentiate the degree of muscle fatigue after entering fatigue with the MMG signal alone, and with the sEMG signal alone; the degree of muscle fatigue after entering fatigue could not be detected earlier, so that the fusion of the two could provide a better analysis of muscle fatigue. A comparison of the peak envelopes of the MPF eigenvalues of the two signals is shown in Figure 9.

Muscle fatigue labels were ascribed for different stages of each muscle, labelling a total of 12 muscles on the left and right sides of the human body. This labelling satisfies two conditions: (1) if at least four muscles are labelled in one stage, the human body state is labelled with the corresponding label; (2) if at least one muscle label is greater than the current human body state label in two stages, the human body state is labelled with the next level label.

Then, we labelled the whole human body state as a result of integrating 16 datasets with 120 data points, and produced three datasets, which were the six-dimensional sEMG dataset consisting of six sEMG signals, the 6-dimensional MMG dataset consisting of six MMG signals, and the 12-dimensional integrated dataset combining the six sEMG signals and the six MMG signals. At this point, we completed the processing of the data and the construction of the dataset. During the subsequent training of the classification model, twenty percent of the available data was randomly extracted as the test set, and the rest was used as the training set.

### 3.3. Construction of a Muscle Fatigue Classification Model

Transformer is a deep learning model architecture for natural language processing and other sequential tasks and was first proposed by Vaswani et al. in 2017 [28]. The Transformer architecture introduces the mechanism of self-attention, which is a key innovation that allows it to excel in processing sequential data.

At this stage, transformer has several models, and one of the applications is the bidirectional encoder representation from transformer (BERT). Li Zhang et al. investigated 15 automated article recommendation methods in the biomedical field. We then empirically evaluated these 15 methods, and the experimental results showed that the BERT model outperformed many existing recommendation methods in most evaluation metrics [29]. Aytuğ Onan proposed a new hierarchical graph-based text classification framework that uses contextual node embedding and BERT-based dynamic fusion to capture complex relationships between nodes in hierarchical graphs and generate more accurate text classifications [30]. In 2021, Francisca Adoma Acheampong discussed various BERT-based models recently proposed by researchers. The review presents their contributions, results, limitations, and datasets used [31]. Rohit Kumar Kaliyar proposed a deep learning method based on BERT by combining different parallel blocks of single-layer deep convolutional neural networks (CNNs) with different kernel sizes and filters with BERT [32].

**Figure 10:**
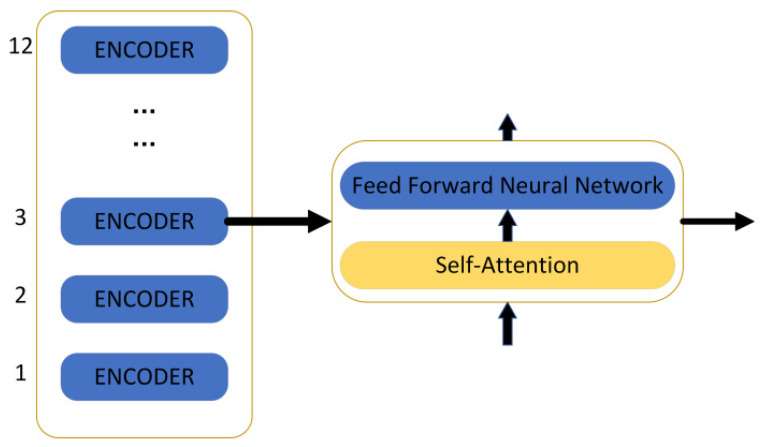
The structure of the BERT model.

Taken together, the BERT model pre-trains deep bidirectional representations from unlabelled texts by carefully weighting the context before and after each layer, and constructs multiple natural language processing tasks, such as text categorisation, automated Q&A, and machine translation, adding only one additional output layer and then fine-tuning it. The BERT model achieved excellent results on a large number of text classification datasets. In this study, we chose the “bert-base-uncased” model, which has 12 hidden layers, a 768-dimensional tensor output, 12 self-attention heads, and a total of 110 M parameters, as shown in Figure 10.

The encoder contains two layers: one is the self-attention layer, and the other is the feed-forward neural network. The self-attention layer helps the current node not only pay attention to the current element but also obtain contextualised connections. The decoder also contains the two network layers mentioned for the encoder, but there is another layer in between these two layers: the attention layer helps the current node to focus on what it needs to. The model needs to perform an embedding operation on the input data, and after the end of embedding, it is input to the encoder layer, and the self-attention sends the data to the feed-forward neural network after processing the data; the computation of the feed-forward neural network can be parallel and the output obtained is input to the next encoder.

The transformer encoder is used because of the self-attention mechanism, so BERT comes with a bidirectional function. Self-attention computes three new vectors, called query, key, and value, which are the result of multiplying an embedding vector with a matrix. In order to improve the computation speed, this study adopted the matrix approach, which directly computes the matrices of query, key, and value, and then directly multiplies the embedded values by the three matrices, multiplies the obtained new matrix Q by K, multiplies it by a constant, performs the softmax operation, and finally multiplies it by the V matrix. This method of determining the weight distribution of the value using the degree of similarity between the query and the key is called scaled dot product attention. The specific calculation formula is shown below.

(4) $$\mathrm{Attention}\left(Q,K,V\right)=\mathrm{softmax}\left(\frac{QK^{T}}{\sqrt{d_{k}}}\right)V$$

**Figure 11:**
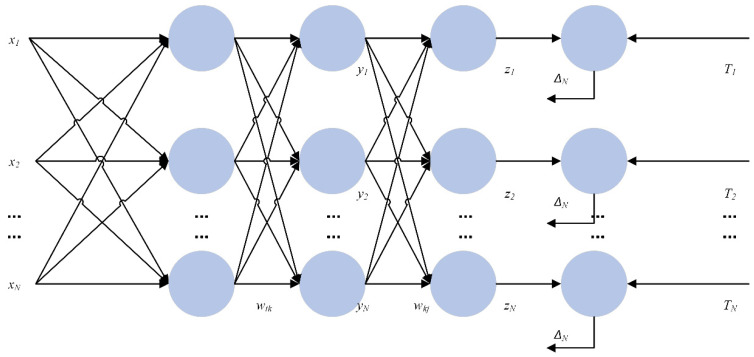
A diagram of the structure of the BP neural network.

After selecting the data processing model, it is also necessary to combine the existing prediction models, and in this study, the error back-propagation neural network was selected to be combined with BERT. The back-propagation neural network, abbreviated as the BP network, is a multilayer neural network with three or more layers, where each layer consists of several neurons. The structure of a BP neural network is shown in Figure 11, which achieves full connectivity between each neuron in the left and right layers, i.e., each neuron in the left layer is connected to each neuron in the right layer, while there is no connection between the top and bottom neurons.

When designing a BP neural network, several aspects should be considered, such as the number of network layers, the number of neurons in each layer, the initial value, and the learning rate. (1) The number of layers of the network: It has been shown that a three-layer BP neural network can realise the mapping from a multi-dimensional unit cube to the mapping of a unit cube, i.e., it can approximate any rational function. (2) The number of neurons in the hidden layers: The accuracy of network training can be improved by increasing the number of neurons in a hidden layer. (3) Choice of initial weights: Since the system is nonlinear, the choice of initial values significantly impacts whether the learning reaches a local minimum, whether converge occurs, and the length of the training time.

In this study, for the dataset dimension of 12 and the data volume size, the parameters of the BP neural network were set to three layers, and the number of neurons in each layer was 180. The study involved parameter optimisation, with the number of layers tested ranging from one to three, and the number of cells tested ranging from 50 to 1000. This set of parameters was adjusted to achieve relatively accurate results. In addition to the selected BP + BERT neural network algorithm, the dataset was also fed into the three BP neural network algorithms, SVM + BERT and SVM + RBF, for classification, and the results of the four algorithms were compared.

**Figure 12:**
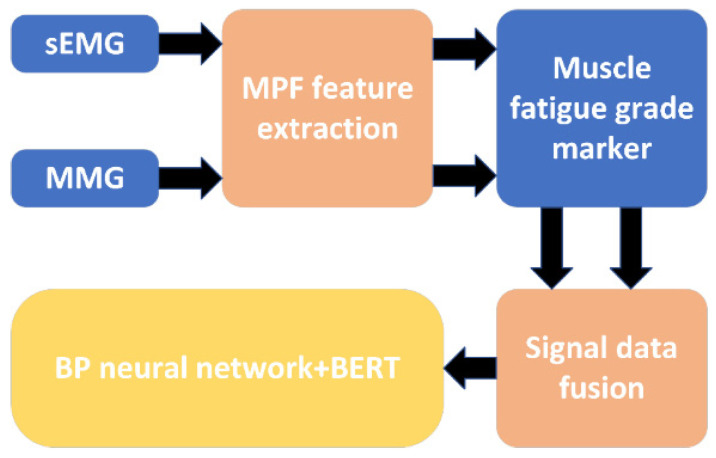
A flowchart of the muscle fatigue classification model.

The construction of the muscle fatigue classification model was completed by importing the fused data into the above data processing model and classification algorithm, and the specific flowchart is shown in Figure 12.

### 3.4. Classification of Evaluation Indicators

Evaluation metrics for pattern classification tasks are used to measure model performance to determine its classification ability and accuracy. In order to compare the muscle fatigue classification results of different algorithms, the confusion matrix, accuracy, precision, recall, and F1 score were chosen as the evaluation indices of the classification results in this study.

The confusion matrix is a tabular tool used to evaluate the performance of a model in a classification problem. It provides a detailed description of the relationship between the model’s classification results and the actual categories and is used to measure the model’s performance metrics, such as accuracy, precision, recall, and F1 score.

The confusion matrix is usually a two-dimensional matrix containing four different classification results:

(1) True Positives (*TP*): These indicate the number of samples in the positive category that the model correctly classifies as positive, i.e., the model correctly identifies positive examples.

(2) True Negatives (*TN*): This indicates the number of samples from negative categories that the model correctly classifies as negative, i.e., the model correctly excludes negative examples.

(3) False Positives (*FP*): This is the number of samples in the negative category that the model incorrectly classifies as positive, i.e., the model incorrectly classifies true negative examples as positive examples.

(4) False Negatives (*FN*): This denotes the number of samples of positive categories that the model misclassifies as negative categories, i.e., the model misclassifies true positive examples as negative examples.

The four elements of the confusion matrix (*TP*, *TN*, *FP*, and *FN*) are the basis for calculating the accuracy, precision, specificity, recall, and F1 score. The confusion matrix provides a clear picture of the classification performance of the model, both in terms of correct classifications and misclassifications, so that the performance of the model can be assessed in different aspects. These metrics have important applications in different tasks and problems, helping to measure the effectiveness of classification models. The formulas for accuracy, precision, specificity, recall, and F1 score are provided below:

Accuracy is the number of samples correctly classified by the model as a proportion of the total number of samples:

(5) $$Accuracy=\frac{TP+TN}{TP+TN+FP+FN}$$

Precision indicates how many of the samples classified as positive categories are actually positive:

(6) $$Precision=\frac{TP}{TP+FP}$$

Recall indicates how many of the samples in the true positive category are correctly classified by the model:

(7) $$Recall=\frac{TP}{TP+FN}$$

The specificity rate indicates the proportion of negative examples identified by the classification out of all negative examples.

(8) $$Specificity=\frac{TN}{TN+FP}$$

The F1 score is the reconciled mean of precision and recall, which is used to combine the accuracy and completeness of the classification:

(9) $$F1\;score=2*\frac{Precision*Recall}{Precision+Recall}$$

The parameters of accuracy, precision, specificity, recall, and F1 score in the confusion matrix accurately reflect the degree of accuracy in predicting different levels of muscle fatigue. The specific, accurate differentiation of fatigue grades can be better applied in subsequent applications.

### 3.5. Results

**Figure 13:**
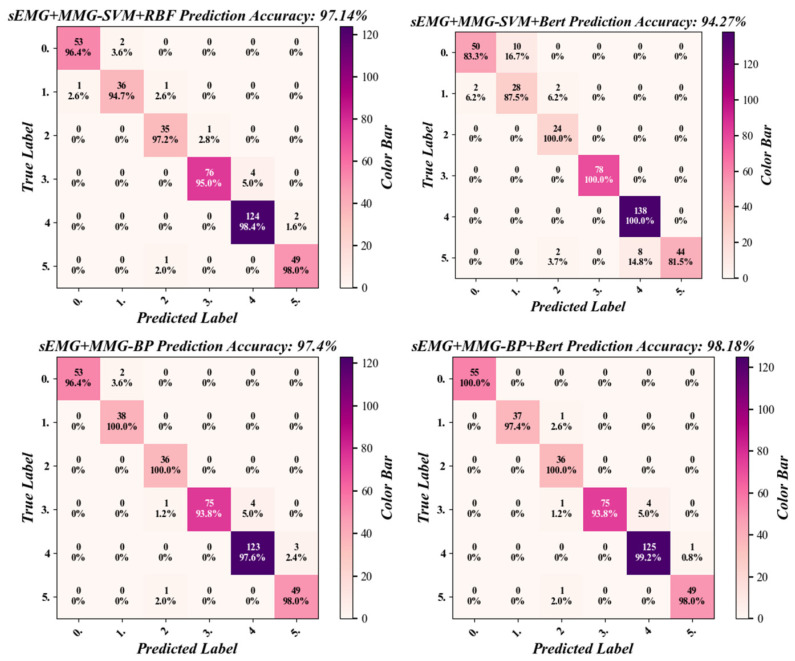
The confusion matrix of the four classification algorithms for the sEMG + MMG dataset.

In this study, for the three established datasets, including the 6-dimensional sEMG dataset composed of six sEMG signals, the 6-dimensional MMG dataset composed of six MMG signals, and the 12-dimensional comprehensive dataset combining six sEMG signals and six MMG signals, the classification comparison of four algorithms was carried out for each of them, respectively. The four algorithms included BP, SVM + BERT, SVM + RBF, and BP + BERT, and each combination was repeated 10 times. The prediction results were subjected to confusion matrix analysis to obtain the confusion matrices of the four algorithms, as shown in Figure 13.

Then the classification metrics were calculated for the confusion matrix and four metrics of precision, recall, specificity, and F1 score. All combinations of the three datasets and the four algorithms were calculated separately to obtain the data shown in Table 3.

As shown in Table 3, which represents the results of one of the muscle fatigue model classifications, the highest precision, recall, specificity, and F1 values in the classification results were all achieved by the BP-BERT algorithm for the sEMG + MMG dataset, with results of 99.12%, 98.52%, 98.18% and 98.79%, respectively.

As shown in Table 4, among the four algorithms, the dataset of sEMG + MMG achieved better performance in the three different datasets with an accuracy above 94%, while the other two datasets had classification accuracies below 91%. In the BP-BERT algorithm, the sEMG + MMG dataset had the highest classification accuracy of 98.01%, while in the SVM + BERT algorithm, the MMG dataset had the lowest classification accuracy of 71.88%.

Table 4 shows that the BP + BERT algorithm excels in classification accuracy. To assess whether there is a statistically significant difference in classification accuracy between BP + BERT and the other algorithms, a two-sample t-test was used. Ten classification accuracies were used as samples, taking the accuracy of the four algorithms in the SEMG + MMG dataset as a reference. The BP + BERT algorithm was solved separately from the other three. Table 5 shows that the *p*-value is significantly lower than the accepted level (α = 0.02).

## 4. Discussion

A comparison of the four algorithms in this study revealed that both the BP and BP + BERT algorithms demonstrate superior performance, with a relatively narrow gap between them. However, no clear advantage emerged across the three datasets. This discrepancy may be attributed to the fact that the two algorithms are well-suited to datasets with similar dimensions; however, the model parameters may not be optimally aligned with the characteristics of the specific dataset. In the combined training of muscle fatigue models, a significant discrepancy in training time was observed, which may be attributed to data dimensions and algorithms that are optimally suited to those dimensions. Additionally, the presence of regression model redundancy, which is a common phenomenon that can prolong training times, may also be a contributing factor.

Compared to Refs. [7,8], this study also incorporated the MMG signal alongside the SEMG signal. This increased the accuracy of the muscle fatigue classification by 1.65–5.1%. This paper also compared the fused dataset with the two independent datasets, showing how this study fused the signals more accurately. In this study, three datasets were collected and compared. It was observed that the classification accuracy of the dataset that combined the two signals was significantly higher than that of the dataset with the separate signals. This may be attributed to the fact that the dimensions of the dataset following the fusion of the two signals increased, thereby containing a greater quantity of information, which is more conducive to muscle fatigue classification.

Compared to Ref. [8], this paper used a more recent classification algorithm, BP + BERT, which is more adaptable than KNN and SVM and can improve classification accuracy continuously by training the model. Among the classification metrics, precision, recall, and F1 score can respond to the accuracy of the classification model by making positive predictions, while the specificity rate indicates the degree of misdiagnosis of the classification model. For the average results of these four metrics, the BP-BERT algorithm has better performance. However, it can be seen in the confusion matrix that the algorithm’s prediction results do not add up in fatigue level 3, probably because of the small amount of data between fatigue levels 2–4, leading to low variability. As the human body changes levels of fatigue faster at this stage, there is no way to collect more data at this stage, and the amount of data in the dataset can be subsequently increased by continuing to add more experiments to improve accuracy at this stage.

This study not only conducted further theoretical research on the parameters in the BP + BERT algorithm but also identified a more suitable parameter through continuous trials. Subsequent research could therefore focus on investigating the parameters in greater detail, with a view to identifying an even more suitable parameter. Furthermore, this study did not conduct a comprehensive theoretical investigation into the algorithmic specifics involved in the selection of the comparative algorithm, which may have resulted in the omission of other potentially suitable algorithms. Furthermore, the continuous updating of algorithms may allow for the emergence of improved algorithms from this study. These findings can be built upon in future research.

The results of the muscle fatigue classification in this study are subject to certain limitations, such as susceptibility to individual differences (e.g., age and physical fitness) and environmental factors (e.g., temperature and type of exercise). Future development will focus on establishing a dynamic classification model that can adapt to different scenarios. Through the measurement and recording of big data, this model could assist sports medicine in developing personalised training programmes and preventing injuries caused by overtraining. The results of muscle fatigue classification can provide a basis for occupational health monitoring and the timely intervention required for chronic muscle fatigue caused by long-term repetitive movements. They can also be used to guide the daily fatigue management of healthy individuals and promote scientific fitness and rest.

## 5. Conclusions

The results of the muscle fatigue model training demonstrate that the classification outcomes of the sEMG and MMG fused dataset, when evaluated using the four algorithms, are superior to those of the other two datasets. This observation suggests that the approach of integrating the sEMG and MMG signals, as employed in this study, is more efficacious. A comparison of the accuracy of the combined datasets and algorithms revealed that the sEMG and MMG fused dataset imported into the BP + BERT algorithm exhibited the highest classification accuracy of 98.18% for the muscle fatigue model. They were also the highest when comparing precision, recall, specificity, and F1 scores, which were 99.12%, 98.52%, 98.18% and 98.79%, respectively.

This study proposes a method of fusing sEMG and MMG signals, which yields superior results. The fusion is implemented using the BP + BERT algorithm, which is then used to construct a muscle fatigue classification model. This model demonstrates enhanced performance, indicating that both the fusion method and the classification model are reasonable and accurate. The proposed approach can be applied in similar studies.

## Figures and Tables

**Table 1:** Name of the selected muscles.

Serial Number	Muscle Name	Position in the Human Body	Sensors Fixed Position	Remarks
1	Vastus medialis	Anteromedial thigh	Sensors were fixed at 80% on the line between the anterior spina iliaca superior and the joint space in front of the anterior border of the medial ligament on the legs.	Lower limb muscle
2	Quadriceps femoris	Anterior middle thigh	Sensors were fixed at 50% on the line from the anterior spina iliaca superior to the superior part of the patella on the legs.	
3	Latissimus dorsi	Back and chest posterolateral	Sensors were fixed to the centre line from the lower chest to the waist and to the humerus behind the armpit.	Waist and abdominal muscles
4	Trapezius	Neck and back	Sensors were fixed to the midpoint of the line from the top of the shoulder to the 7th cervical vertebra.	Upper limb muscles
5	Deltoid	Upper shoulder side	Sensors were fixed 1 finger’s width in front of the shoulder crest	
6	Biceps brachii	Anterior upper arm	Sensors were fixed parallel to the line between the acromion and the ulnar fossa	

**Table 2:** Classification of muscle fatigue levels.

Labelling of Muscle Fatigue Levels	Range of MPF Changes	Levels of Muscle Fatigue
0	Before the MPF signal peaks	Muscle is not fatigued
1	MPF signal grows to peak	Fatigue transition state
2	MPF signal peaks down to 5 per cent	
3	MPF signal peaks down to 10 per cent	
4	MPF signal peaks down to 15 per cent	
5	MPF signal peaks down to 20 per cent	Muscle fatigue

**Table 3:** The classification results from one of the muscle fatigue models.

Signal Composition of the Dataset	Name of the Algorithm	Classification Accuracy	Precision	Recall	Specificity	F1 Score
sEMG	SVM + RBF	80.73%	90.17%	85.16%	83.73%	85.86%
	SVM + BERT	77.01%	83.24%	84.35%	76.9%	82.84%
	BP	89.11%	91.97%	92.01%	90.6%	91.71%
	BP + BERT	86.41%	92.23%	92.12%	86.07%	91.82%
MMG	SVM + RBF	80.73%	84.16%	74.62%	77.37%	75.94%
	SVM + BERT	71.88%	66.98%	70.38%	71.62%	67.31%
	BP	81.56%	86.58%	81.73%	83.66%	82.69%
	BP + BERT	84.58%	84.54%	81.55%	81.99%	81.65%
sEMG + MMG	SVM + RBF	97.14%	98.14%	97.22%	97.12%	97.67%
	SVM + BERT	94.27%	93.69%	95.14%	94.12%	94.21%
	BP	97.34%	98.72%	98.35%	97.41%	98.5%
	**BP + BERT**	**98.10%**	**99.12%**	**98.52%**	**98.18%**	**98.79%**

**Table 4:** Results of muscle fatigue classification model accuracy.

Signal Composition of the Dataset	Name of the Algorithm	Classification Accuracy (Mean ± Standard Deviation)
sEMG	SVM + RBF	83.59 ± 1.86%
	SVM + BERT	76.26 ± 1.39%
	BP	90.10 ± 2.13%
	BP + BERT	86.77 ± 1.09%
MMG	SVM + RBF	77.68 ± 3.05%
	SVM + BERT	71.00 ± 1.51%
	BP	83.73 ± 2.35%
	BP + BERT	84.01 ± 1.94%
sEMG + MMG	SVM + RBF	97.00 ± 0.93%
	SVM + BERT	93.87 ± 0.70%
	BP	97.49 ± 0.40%
	**BP + BERT**	**98.01** ± 0.84%

**Table 5:** Results of two-sample T-test for different algorithms.

Method	T-Statistic	*p*-Value
**BP + BERT**	/	/
SVM + RBF	3.4805736	0.0083126
SVM + BERT	3.2689005	0.0113748
BP	10.174702	7.4575106 × 10^−6^

## Data Availability

The datasets used or analysed during the current study are available from the corresponding author on reasonable request.
